# Revitalizing Postoperative Pain Management in Enhanced Recovery After Surgery via Inter-departmental Collaboration Toward Precision Medicine: A Narrative Review

**DOI:** 10.7759/cureus.59031

**Published:** 2024-04-25

**Authors:** Nobuyasu Komasawa

**Affiliations:** 1 Community Medicine Education Promotion Office, Faculty of Medicine, Kagawa University, Miki-cho, JPN

**Keywords:** precision medicine, inter-departmental collaboration, complication, safety, postoperative pain

## Abstract

This narrative review explores the crucial aspects of postoperative pain management within the framework of Enhanced Recovery After Surgery (ERAS). It emphasizes the significance of effective and secure pain management, highlighting its impact on patient well-being, surgical outcomes, and hospital stays. The inadequacy of perioperative pain relief increases the risk of persistent postoperative pain, emphasizing the need to challenge the notion that pain is expected after surgery. The goals of postoperative pain management extend beyond mere relief, encompassing comfortable sleep, pain-free rest, and liberation from pain during initial recovery. Inadequate pain management can lead to complications such as heightened postoperative bleeding and an increased risk of thrombosis. The review delves into various analgesic methods, their complications, and safety measures. ERAS programs, focused on reducing complications and medical costs, emphasize the importance of judicious postoperative pain management and active rehabilitation. The review discusses complications associated with analgesic methods like opioids, epidural analgesia, and adjuvant analgesics. Collaboration within the perioperative management team is crucial for effective postoperative pain relief. Interdepartmental collaboration is essential for evaluating surgical procedures, analgesic methods, and crisis management strategies. The review concludes by integrating precision medicine into postoperative pain management, emphasizing the potential of genetic information in assessing pain sensitivity. It underscores the importance of inter-departmental collaboration and information gathering for the successful implementation of precision medicine tailored to each facility's perioperative management systems. Additionally, the impact of artificial intelligence (AI) on preoperative risk assessment and innovative monitoring techniques is discussed, paving the way for the advancement of precision medicine in postoperative pain management.

## Introduction and background

Postoperative analgesia is crucial for the recovery and improvement of the quality of life for patients post-surgery. The pain associated with surgery not only causes discomfort for patients but also has physiological implications [1]. In addressing these challenges, the adoption of postoperative analgesia becomes essential.

When postoperative pain is effectively controlled, patients experience relief from discomfort, leading to an enhancement in their overall quality of life. This, in turn, allows patients to concentrate more effectively on the recovery process. Furthermore, the improvement in surgical outcomes, coupled with a reduction in complications and recovery time, is observed.

Postoperative analgesia plays a significant role in mitigating physiological impacts. Unmanaged pain can adversely affect the circulatory and respiratory systems, placing a burden on the overall stability of the patient's body. Through analgesic interventions, these physiological impacts can be minimized.

Preventing chronic pain is another critical objective of postoperative analgesia. It has been suggested that inadequate management of postoperative pain increases the risk of developing chronic pain [2,3]. Prevention of such chronic pain not only maintains the quality of life (QOL) in patients but also ensures the effective utilization of medical resources.

Enhancing patient satisfaction is also a component of the role played by postoperative analgesia. Effective management of postoperative pain contributes to an improved patient experience, fostering trust and rapport between healthcare providers and patients. Thus, postoperative analgesia is indispensable for post-surgical patient care, impacting various aspects such as patient comfort, physiological stability, surgical outcomes, prevention of chronic pain, and enhancement of quality of perioperative life.

In this narrative review, we discuss the significance of inter-department postoperative pain management in the digital health medical environment including precision medicine in postoperative pain.

## Review

Postoperative pain management from the viewpoint of enhanced recovery after surgery (ERAS)

The pursuit of effective postoperative pain management that not only proves effective but also remains free from complications is a paramount ideal in the realm of healthcare. This narrative review embarks on a thorough exploration of the significance of postoperative pain and potential complications and underscores the paramount importance of fostering inter-departmental collaboration to realize effective and secure pain management strategies.

**Figure 1:**
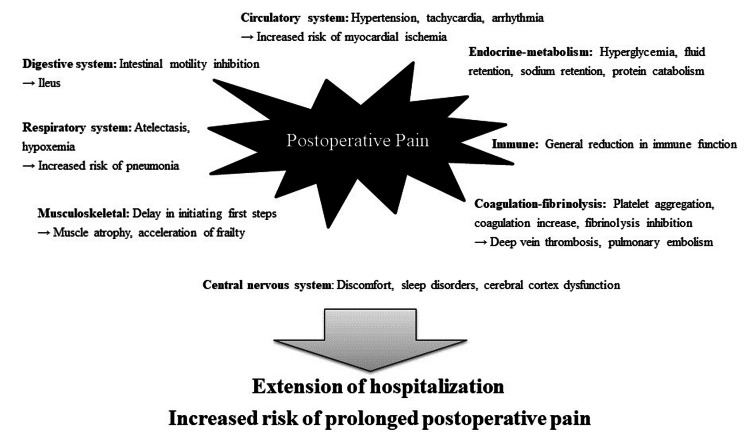
Various physiological effects of postoperative pain on the living body. Image credit: Nobuyasu Komasawa.

Postoperative pain transcends the realm of mere patient discomfort, exerting profound effects on the respiratory and circulatory systems, as elaborated in Figure 1. These impacts extend beyond individual well-being to influence surgical outcomes and the duration of hospital stays. The inadequacy of pain relief during the relatively brief perioperative period significantly heightens the risk of transitioning into persistent postoperative pain [1,2], often necessitating prolonged treatment interventions [3]. Consequently, dismissing the notion that *pain is expected after surgery *becomes imperative for comprehensive patient care.

The primary objective of postoperative pain management extends beyond mere pain relief; it includes the attainment of the ability to sleep comfortably. Subsequently, the second goal is the absence of pain during rest, and the aspirational third goal revolves around liberating patients from pain during the initial postoperative steps and rehabilitation. The ramifications of insufficient postoperative pain management are multifaceted, impacting not only patient suffering but also potentially contributing to heightened postoperative bleeding attributable to increased blood pressure and an augmented risk of thrombosis due to limitations in daily activities [4,5]. Furthermore, it is crucial to acknowledge that all postoperative pain management modalities wield both positive effects and potential complications, necessitating meticulous consideration in their application.

In short, the comprehensive understanding of the multifaceted nature of postoperative pain and its implications emphasizes the need for a nuanced and collaborative approach. Inter-departmental collaboration becomes the linchpin in achieving effective and secure postoperative pain management, aligning with the broader objectives of ensuring patient well-being and optimal outcomes in the intricate landscape of healthcare.

In recent years, there has been a notable accumulation of evidence surrounding ERAS programs [6,7]. These programs are strategically designed with overarching goals that encompass the reduction of postoperative complications, enhancement of safety protocols, shortening hospital stays, and minimizing medical costs. The successful implementation of ERAS relies on two pivotal components: judicious postoperative pain management and active rehabilitation. Consequently, the attainment of safe and effective postoperative pain management systems stands as a linchpin in the realization of comprehensive ERAS objectives [8,9].

The collective anticipation for advanced postoperative pain management systems is considerable, aligning seamlessly with the broader ERAS framework and its associated benefits for both patients and healthcare systems alike. As the evidence surrounding ERAS programs continues to grow, the focus on judicious postoperative pain management becomes increasingly central to the success of these programs. Advanced pain management not only contributes to enhanced patient comfort but also plays a pivotal role in facilitating the swift recovery emphasized by ERAS protocols.

The dynamic interplay between ERAS programs and postoperative pain management underscores the evolving landscape of surgical care. Anticipating and embracing advancements in postoperative pain management aligns with the overarching objectives of ERAS, contributing to improved patient outcomes and resource utilization. The synergy between these components reflects a comprehensive approach to perioperative care, emphasizing the importance of integrating advanced pain management strategies into the broader context of enhanced recovery initiatives.

Complications and safety measures of various analgesic methods

Here, a comprehensive overview of complications and corresponding safety measures associated with various analgesic methods employed in postoperative pain management is summarized. Fentanyl, a frequently employed opioid for postoperative pain in the perioperative period, exhibits robust analgesic efficacy against visceral pain. However, its use comes with potential complications, including respiratory suppression, nausea, constipation, and ileus [10]. Caution is particularly warranted in cases where fentanyl is frequently co-administered with antiemetic drugs like droperidol, as this combination may precipitate extrapyramidal side effects [11].

Continuous Epidural analgesia, a technique involving the placement of a catheter in the epidural space for the continuous administration of local anesthetics or opioids, demonstrates notable efficacy in providing selective analgesic effects [12,13]. However, vigilance is imperative as excessive concentrations or doses of local anesthetics may lead to complications such as hypotension, motor paralysis, and spinal cord infarction [14]. Continuous monitoring is also indispensable to forestall potential complications arising from hematoma formation or infection, which could result in spinal cord compression [15].

The advent of ultrasound technology has revolutionized the visualization of peripheral nerve anatomy, facilitating the administration of peripheral nerve blocks (PNBs). Despite the advantages, complications such as nerve damage or local anesthetic toxicity necessitate early detection and management. While evidence on PNB utility is accumulating [16,17], there is a need for enhancements in the documentation of neurologic complications following regional anesthesia. Establishing standardized definitions and consistent time points for assessing these outcomes will facilitate the accurate determination of incidence rates and better quantify the issue across different PNBs. While the optimal objective is to position a needle outside the epineurium but as close to the nerve as possible, inadvertent intraneural injections are common during both nerve stimulator (NS)- and ultrasound (US)-guided PNBs, yet they may not invariably result in nerve injury. Prior animal and human research has identified potential risk factors, but future investigations should employ rigorous scientific methodologies to identify and categorize the diverse risk factors significant for neurologic outcomes. Improving study designs and statistical methods to address the multitude of sources, challenges in data collection, and variations in statistical analyses may enhance the evidence concerning crucial risk factors for neurologic complications post-PNB.

Adjuvant analgesics, including nonsteroidal anti-inflammatory drugs (NSAIDs) and acetaminophen, prove effective against somatic pain, particularly incisional pain. In certain surface surgeries, these adjuvant analgesics alone may suffice to achieve adequate analgesia. Scheduled administration of adjuvant analgesics is also implemented to mitigate complications associated with continuous fentanyl intravenous infusion or continuous epidural analgesia [18]. However, exercising caution is imperative, as NSAIDs may induce kidney damage, and acetaminophen may lead to liver damage, underscoring the importance of continuous monitoring [19].

In summary, perioperative healthcare practitioners should possess a nuanced understanding of these basic complications associated with postoperative pain, fostering interprofessional insights into the considerations and safety measures integral to the deployment of various analgesic methods in the intricate landscape of postoperative pain management. This comprehensive knowledge forms the foundation for a collaborative and vigilant approach to ensuring patient safety and optimal pain relief in the perioperative period.

Perioperative management team as a safety net for postoperative pain management

Inadequate pain control not only impacts patient comfort but also influences surgical outcomes and hospital stays. Persistent postoperative pain risk increases when perioperative pain relief is insufficient, necessitating extended treatment interventions. Dismissing the idea that pain is expected after surgery becomes crucial for comprehensive patient care. ERAS programs, to reduce complications and costs while improving safety, have gained prominence [18]. ERAS's success relies on judicious postoperative pain management and active rehabilitation. Recent years have seen a growing body of evidence supporting ERAS, aligning with broader expectations for advanced postoperative pain management. Although various analgesic methods present complications and safety measures, we can minimize the negative effects with a team approach. Utilizing a team approach, we can detect respiratory suppression by opioids or low blood pressure caused by epidural analgesia. Interdisciplinary collaboration within the perioperative management team is essential to ensuring effective and secure postoperative pain relief. The team, comprising anesthesiologists, surgeons, nurses, and physical therapists, plays a crucial role in evaluating postoperative pain. Training programs should establish shared understanding and goals, fostering collaboration and communication among team members. Interdepartmental collaboration is essential for optimal postoperative pain relief, requiring a unified goal that transcends individual departmental boundaries.

The safety landscape of healthcare in Japan is significantly influenced by principles derived from the aviation and manufacturing industries, emphasizing *safe protocols*, *monitoring*, and *response to emergencies *[19]. These principles serve as the foundation for ensuring the safety of healthcare practices. However, the intricate nature of postoperative pain management introduces complexities such as variations in pain sensitivity, diverse surgical procedures, and fluctuations in intensity during the recovery period. These challenges underscore the need for more extensive and nuanced approaches that complement the foundational principles.

Therefore, there is a critical imperative to foster resilience within perioperative management teams across diverse hospitals through collaborative initiatives [20,21]. This involves promoting a culture of adaptability and responsiveness within the healthcare system, aligning it with the high standards set by industries with proven safety records.

To ensure the delivery of effective and secure postoperative pain management, it becomes imperative to establish a comprehensive safety net facilitated by interdisciplinary collaboration within the perioperative management team [22,23]. Their collective expertise and collaboration contribute to a holistic approach to patient care, ensuring that pain management strategies are not only effective but also tailored to individual patient needs.

The initiation of developing the perioperative management team in the domain of postoperative pain management should be approached by cultivating a shared understanding and common goals among all healthcare providers involved, both within the operating room and in the ward [24,25]. This shared understanding extends to the importance of postoperative pain management and the safety considerations associated with it.

This shared understanding lays the groundwork for a more comprehensive and interconnected approach to postoperative pain management, ensuring that the entire perioperative team is aligned in providing optimal care for patients undergoing surgical procedures. By emphasizing collaboration and a shared commitment to safety, the healthcare system can enhance its ability to address the intricacies of postoperative pain management and ultimately improve patient outcomes.

Inter-departmental collaboration for the postoperative pain management team

**Figure 2:**
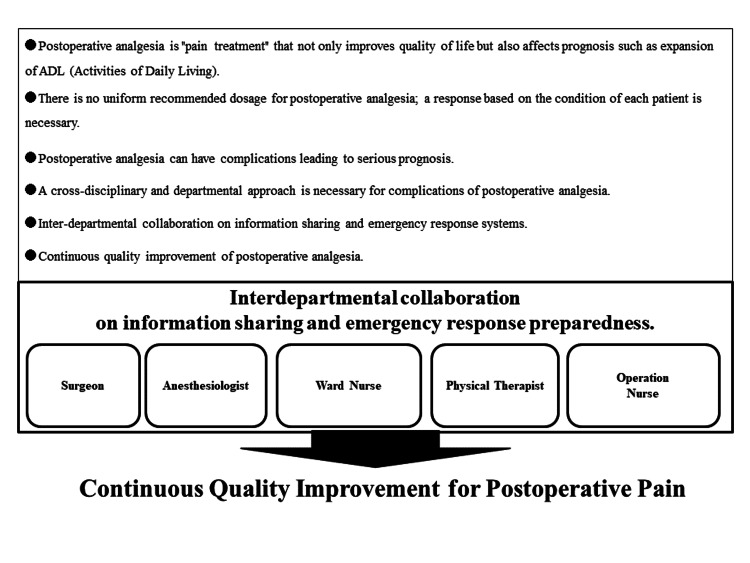
Diversity in postoperative pain and the necessity of a coordinated postoperative pain system. Image credit: Nobuyasu Komasawa.

In the context of Japan's healthcare system, a noteworthy development unfolded during the medical fee revision of 2022 with the introduction of the *Postoperative Pain Management Team Additional Fee*. This fee structure is contingent upon the execution of postoperative pain management after general anesthesia, orchestrated by a multidisciplinary team [26,27]. It is essential to recognize that this team extends beyond anesthesiologists to include nurses, pharmacists, and clinical engineering technologists, underscoring the integral role played by the perioperative management team in ensuring comprehensive and effective postoperative pain relief. Figure 2 elucidates instances of shared understanding and goals related to the significance and safety considerations of postoperative pain, serving as a foundational framework for fostering effective collaboration and communication.

Facilitating optimal postoperative pain relief through collaborative efforts within the perioperative management team necessitates that all team members possess a nuanced understanding of the significance, methodologies, and potential complications associated with postoperative pain relief. To achieve this, dedicated off-the-job discussions are imperative to foster an environment conducive to open discourse, free from the constraints of clinical duties, and should incorporate insights from diverse disciplines. Engaging in collaborative discussions spanning multiple disciplines, including surgeons, anesthesiologists, and various specialists, becomes essential for evaluating the efficacy and potential complications of diverse surgical procedures and analgesic methods, as well as formulating crisis management strategies.

Educational initiatives targeting the perioperative management team with a focus on postoperative pain relief should extend beyond medical staff solely within the operating room. Surgeons, ward nurses, and physical therapists must also be included in these programs to ensure a comprehensive understanding and collaborative approach. The incorporation of simulation training, utilizing problem-based learning methodologies and involving multiple disciplines within the hospital setting, stands as a promising avenue for continuous quality improvement in postoperative pain management [28,29].

In the realm of postoperative pain management, adopting an integrated approach by confining responsibilities to specific departments, such as attributing the entirety of postoperative pain management to anesthesiologists or assigning adjustment duties solely to the ward, is deemed inappropriate. Achieving effective and safe postoperative pain relief for patients requires a unified goal, transcending individual departmental boundaries. The realization of this common objective necessitates robust inter-departmental collaboration between the operating room and the ward, seamlessly integrated as part of the perioperative management team [30]. The promotion of heightened awareness regarding interdepartmental collaboration within the perioperative management team is anticipated to serve as a catalyst for enhancing the efficacy and safety of postoperative pain relief.

Revolutionizing anesthesia medicine: the unleashed potential of artificial intelligence (AI) integration for personalized patient care

The current state of AI, equipped with advanced analytical capabilities through deep learning, has initiated innovative changes, extending its influence on perioperative management. Thus, anesthesiologists navigating the AI era are required to cultivate nontechnical skills, specifically clinical judgment skills related to risk management. By adeptly replicating these higher-order cognitive functions, AI emerges as a significant contributor to various facets of medical practice, including research, diagnosis, treatment planning, and predictive modeling. It is essential for healthcare professionals to recognize the transformative potential of AI in perioperative management and to develop the necessary skills to leverage its capabilities responsibly and effectively. AI's rapid integration of information continues to exert a profound and sustained impact on clinical settings, and the realm of anesthesia medicine is certainly no exception. Among the multifaceted effects that AI is beginning to imprint on anesthesia medicine, the foremost two dimensions encompass (1) bolstering support for preoperative risk assessment and (2) fostering innovative monitoring capabilities through seamless information integration [31].

Beyond the realms of preoperative risk assessment and monitoring, the expansive potential of AI becomes evident in its ability to harmonize and dissect past patient data (e.g., anesthesia or procedural sedation history) alongside intricate pathophysiological insights, thereby heralding a new era of personalized anesthesia management, inclusive of perioperative pain management leading to active rehabilitation [32,33]. In other words, there is a possibility of further ERAS improvement.

Delving into the domain of preoperative risk assessment support, AI emerges as a pivotal player, proffering invaluable information for the meticulous pre-assessment of patients. Essentially, by distilling electronic medical records, including preoperative test results, AI stands poised to furnish optimal recommendations for anesthesia management, extending to postoperative analgesia. As an anesthesiologist crafts a treatment plan for a patient, AI lends its prowess by presenting the latest clinical guidelines and medical literature, facilitating decision-making through suggestions on anesthesia techniques, and highlighting medications contraindicated for specific cases [34].

The convergence of preoperative information and monitoring, encapsulating patient conditions and pathophysiology, holds transformative promise for personalized anesthesia management [35]. As the reservoir of big data swells during the perioperative period, the meticulous verification of individual patient attributes such as age, gender, test results, pathophysiology, and surgical procedures becomes conceivable. This could pave the way for the development of anesthesia protocols tailored to the nuanced needs of each patient and their unique condition [36]. 

Furthermore, the integration of patient test values with historical anesthesia and sedation data may unlock possibilities for further mitigating risks associated with postoperative analgesia, with AI proffering recommendations that transcend the confines of current clinical trials or drug mechanisms.

Already, numerous facilities have embraced electronic anesthesia records alongside electronic medical records. The judicious application of AI's advanced analytical capabilities is anticipated to propel the frontiers of personalized anesthesia management significantly. From a broader perspective, the synergy of AI and telemedicine holds the promise of providing robust support for anesthesia and postoperative analgesia in the context of regional healthcare settings [37]. As these technologies continue to evolve, their potential impact on refining and individualizing anesthesia and pain management practices is expected to expand even further.

Advancing precision medicine in postoperative pain management: an inter-departmental collaboration for tailored care

Precision medicine, heralded as a revolutionary approach, is defined by its commitment to crafting personalized medical strategies based on the intricate interplay of an individual patient's genetic makeup, environment, and lifestyle. This marks a distinct departure from conventional treatment paradigms [38]. Unlike the one-size-fits-all approach of general treatment methods and medical policies, precision medicine meticulously tailors treatments, considering the unique characteristics of each patient.

The primary objective of precision medicine extends beyond the mere provision of medical care; it strives to discover optimal treatment methods finely tuned to the individual patient. This approach seeks to maximize the effectiveness of treatment while minimizing potential side effects, presenting a paradigm shift from traditional medical practices [39]. Its application is particularly pronounced in the realm of cancer treatment, where ongoing efforts involve the exploration of effective drug therapies for specific cancers through the genomic analysis of cancer cells. As precision medicine continues to evolve, its potential may extend beyond cancer to encompass a wide array of diseases, ushering in a new era of more personalized healthcare.

Importantly, precision medicine transcends specific medical domains and finds relevance in perioperative care and postoperative pain management. While precision medicine, with its initial focus on individual genomic information, has been primarily associated with cancer treatment, it also holds promise in addressing pain sensitivity, including postoperative pain. Recent advancements in identifying genetic polymorphisms linked to pain suggest that, in the future, assessing pain sensitivity through preoperative tests might be feasible [40,41].

**Figure 3:**
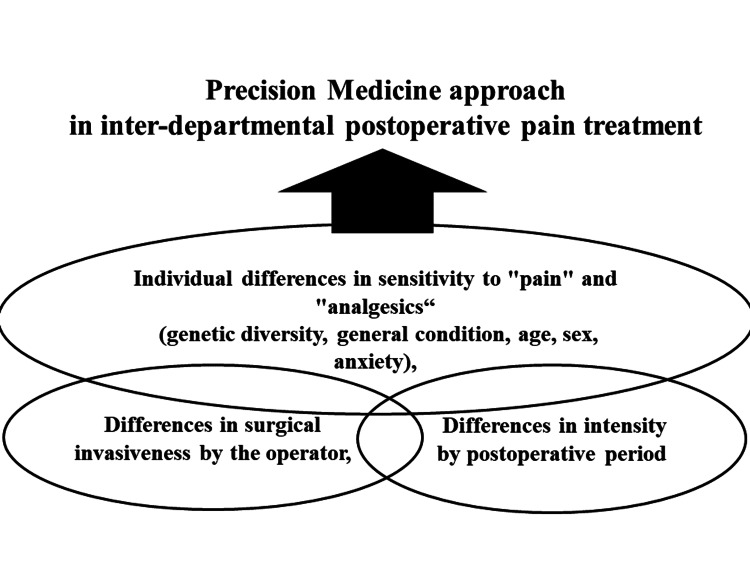
Precision medicine approach in inter-departmental postoperative pain treatment. Image credit: Nobuyasu Komasawa.

Furthermore, the integration of variables such as the size of surgical incisions and facility-specific postoperative management systems can contribute to the development of precision medicine in postoperative pain management. In essence, collaborative information gathering across departments emerges as the linchpin for pioneering precision medicine in postoperative pain management tailored to the diverse perioperative management systems of individual facilities (Figure 3). This collaborative approach ensures that precision medicine is seamlessly integrated into the perioperative care continuum, optimizing outcomes for patients undergoing surgical procedures.

The rapid integration of information facilitated by AI continues to exert a profound and consistent influence on clinical settings, marking a transformative era in the field of anesthesia medicine. AI's impactful contributions are particularly pronounced in two crucial areas: (1) providing robust support for preoperative risk assessment and (2) introducing innovative monitoring techniques through comprehensive information integration [42]. However, beyond these foundational aspects, a tantalizing prospect emerges as AI assimilates and scrutinizes historical patient data, encompassing anesthesia or procedural sedation history, and delves into pathophysiological information. This not only holds the promise of enhancing anesthesia medicine but also positions AI to play a pivotal role in advancing precision medicine to the forefront of postoperative pain management [43,44].

As AI becomes more adept at processing and analyzing vast amounts of patient data, it opens avenues for a deeper understanding of individual responses to anesthesia and pain management. By incorporating historical patient information and delving into the intricacies of pathophysiological factors, AI has the potential to refine and personalize postoperative pain management strategies. In other words, precision medicine combined with AI can provide the best ERAS program for each patient. This evolution is not only about improving the immediate perioperative experience but also aligns with the broader goals of precision medicine, which seeks to tailor medical interventions based on individual characteristics.

The integration of AI into postoperative pain management holds promise in optimizing treatment plans by considering a patient's unique history, response patterns, and potential risk factors. This goes beyond conventional approaches, paving the way for a more nuanced and patient-centric paradigm. The collaborative synergy of AI with precision medicine creates a dynamic landscape that has the potential to revolutionize how postoperative pain is understood, assessed, and managed.

In essence, the ongoing advancements in AI's role in anesthesia medicine extend beyond immediate perioperative concerns, providing a foundation for a more personalized and sophisticated approach to postoperative pain management. As technology continues to evolve, the synergistic integration of AI and precision medicine is poised to shape the future of healthcare, ensuring that patient care becomes increasingly tailored to individual needs and characteristics.

## Conclusions

Effective postoperative pain management is crucial for optimizing patient outcomes and facilitating a smooth recovery process. ERAS programs underscore the significance of judicious pain management as a pivotal aspect of perioperative care. Collaboration among healthcare providers from various specialties is essential for addressing the multifaceted nature of postoperative pain and mitigating potential complications. While various analgesic methods offer benefits, they also present risks that require careful consideration and monitoring. Interdisciplinary collaboration within the perioperative management team is vital for assessing pain levels, identifying complications, and implementing appropriate interventions. By fostering a shared understanding and common goals among team members, healthcare providers can ensure that postoperative pain relief strategies are effective and safe.

The integration of AI and precision medicine holds promise for further enhancing postoperative pain management. AI's advanced analytical capabilities can personalize treatment plans based on individual patient characteristics and needs, improving preoperative risk assessment and optimizing pain relief strategies. This innovative approach has the potential to transform postoperative pain management, leading to better outcomes and patient satisfaction. Collaborative efforts and the adoption of innovative technologies are essential for advancing postoperative pain management and enhancing the overall success of surgical procedures. Continued interdisciplinary collaboration and the integration of cutting-edge solutions will drive improvements in perioperative care and patient experiences.
